# Development and Validation of a Standardised Genomic Tool for Conservation Management of the Koala (*Phascolarctos cinereus*)

**DOI:** 10.3390/ani15233375

**Published:** 2025-11-21

**Authors:** Lily F. Donnelly, Shannon R. Kjeldsen, Matthew J. Lott, Kellie Leigh, Matthew A. Field, Ira R. Cooke, Belinda R. Wright, Kyall R. Zenger

**Affiliations:** 1 College of Science and Engineering, James Cook University, Townsville, QLD 4811, Australia; shannon.kjeldsen@oist.jp (S.R.K.); matt.field@jcu.edu.au (M.A.F.); ira.cooke@jcu.edu.au (I.R.C.); kyall.zenger@jcu.edu.au (K.R.Z.); 2 Marine Climate Change Unit, Okinawa Institute of Science and Technology, Onna-son 904-0945, Okinawa, Japan; 3 Australian Centre for Wildlife Genomics, Australian Museum, Sydney, NSW 2010, Australia; matthew.lott@australian.museum (M.J.L.); belinda.wright@sydney.edu.au (B.R.W.); 4 Science for Wildlife, Sydney, NSW 2785, Australia; kellie@scienceforwildlife.org; 5 Sydney School of Veterinary Science, University of Sydney, Camperdown, NSW 2006, Australia

**Keywords:** conservation genomics, host-pathogen interactions, population structure and diversity, Single Nucleotide Polymorphism (SNP), standardised assay

## Abstract

Koalas (*Phascolarctos cinereus*) are threatened by habitat loss, fragmentation, and population isolation, increasing the risk of inbreeding and extinction. Genomic tools are valuable for guiding management decisions, and a standardised tool genomic is the most effective approach. In this study, an integrated genomic SNP assay was developed and validated as a comprehensive monitoring tool for koala conservation. The panel unifies SNP markers from previous approaches (DArTseq, exon-capture, whole-genome sequencing) into a standardised platform and incorporates novel fitness-related loci linked to immunity, thermoregulation, diet, and reproduction, alongside pathogen targets such as koala retrovirus (KoRV) and koala papillomavirus (KoAA). The assay was validated across key conservation applications, including population diversity and differentiation, parentage assignment, sex determination, provenance testing, and pathogen screening, using a variety of sample types (blood, tissue, swabs, scat), from previously tested populations across the distribution. A total of 3358 informative SNPs were identified, including 210 high-confidence outliers associated with immune and stress-response functions, indicating strong potential to capture adaptive variation. By integrating existing genomic resources with new adaptive and predominant pathogen loci, this cost-effective, standardised assay provides a unifying genomic framework for koala management, supporting applications from veterinary diagnostics to long-term monitoring under the National Koala Recovery Plan.

## Simple Summary

Koalas are one of Australia’s most iconic animals, yet face serious threats including habitat loss, climate change, and infectious diseases. Protecting them requires not only saving habitat but also understanding their genetic diversity, ensuring gene flow, and monitoring disease risks. Until now, genetic studies of koalas have used a variety of methods, making it complex to compare results across time and populations. In this study, the first standardised genomic test for koalas was developed, bringing together information on population diversity, adaptation, and multiple major pathogens into a single tool. The study shows the genotyping assay works with many sample types, including ‘non-invasive’ such as scat and swabs, making it widely accessible for research, management and veterinary use. The tool can be used for applications such as identifying family relationships, sex, population origins, and genetic markers in ecologically and biologically important candidate genes. It also detected the presence of important pathogens. By creating a single, affordable and standardised test, this study provides a common baseline for future koala monitoring projects. This will help conservation managers, researchers, and wildlife carers to work together more effectively and make evidence-based decisions to safeguard the long-term survival of koalas across their range.

## 1. Introduction

The koala (*Phascolarctos cinereus*) is an internationally iconic marsupial, endemic to Australia [1]. An arboreal folivore, koalas feed almost exclusively on *Eucalyptus* leaves, restricting their distribution to forests and woodlands along eastern and south-eastern Australia [2,3]. Historically, koala populations experienced severe declines due to extensive hunting for fur in the late 19th and early 20th centuries [4,5,6,7] following European settlement, and have since been further impacted by widespread habitat loss from land clearing and urban development [8,9]. Subsequent management interventions- translocations to island ‘safe havens’, particularly the southern states often involved few founders and further reduced genetic diversity, amplifying bottleneck effects [6,8,10,11,12,13]. Together with biogeographic barriers, these events have shaped the genetic structure of contemporary populations, including severe bottlenecks and reduced diversity in some populations, particularly southern Australia [13,14,15,16]. This has led to classification of *Vulnerable* on the International Union of Conservation of Nature (IUCN) Red List and Nationally, under the Environmental Protection and Biodiversity Conservation (EPBC) Act; listed as *Endangered* in Queensland (QLD), New South Wales (NSW) and the Australian Capital Territory, although not listed in Victoria (VIC) and South Australia (SA) [1,17,18].

Today, koalas remain threatened by ongoing habitat fragmentation, vehicle collisions, dog attacks, bushfires, and climate change, including rising temperatures and increasing aridity [2,19,20,21,22]. Infectious diseases further contribute to morbidity and mortality, most notably *Chlamydia*, which is exacerbated by koala retrovirus (KoRV) infection, also suspected to play a role in neoplasia [23,24,25]. Small and isolated populations are particularly vulnerable to inbreeding, genetic drift, and reduced adaptive potential, which can accelerate extinction risk [26,27,28,29,30,31]. These challenges highlight the need for integrated management approaches that combine ecological and genetic data.

Genetic tools are critical for guiding conservation actions, providing insights into population structure, diversity, inbreeding, connectivity, and adaptive variation [30,32,33,34,35]. Single Nucleotide Polymorphism (SNP) panels and genome-wide association studies (GWAS) have proven valuable in informing conservation —for example, the Iberian lynx (*Lynx pardinus*) [36], Delta Smelt (*Hypomesus transpacificus*) [37], Squirrel (*Petaurus norfolcensis*) and Mahogany (*Petaurus gracilis*) gliders [38] and northern quoll (*Dasyurus hallucatus*) [37]. Historically, koala genetic studies have utilised minisatellites [39,40,41], Randomly Amplified Polymorphic DNA (RAPD) markers [42,43], mitochondrial DNA (mtDNA) [8,12,43,44,45] and microsatellites [10,14,46,47,48,49,50]. Recently, next-generation sequencing (NGS) technologies have greatly improved capacity to explore both neutral and adaptive variation, including Diversity Arrays Technology—sequencing (DArTseq) [13,51], exon capture [16,52], and whole-genome sequencing (WGS) [53,54,55] in koalas. Interpreting and consistently applying such data in conservation decision-making, however remains challenging for managers, highlighting the value of a standardised tool.

An effective conservation genomic tool should provide easy-to-interpret, biologically meaningful information, be cost-efficient and broadly compatible with both invasive and non-invasive sample types to ensure maximum applicability [32,56]. Neutral markers remain essential for assessing genetic diversity, connectivity, and long-term viability, whereas adaptive loci provide insights into local adaptation and resilience to threats such as disease and climate change [33,57,58,59]. Core applications extend to sex determination, valuable for joeys, archived material, or non-invasive samples where individuals are not directly observed; parentage assignment, managing pedigrees and minimising inbreeding in captive or semi-managed populations; and provenance testing, enabling informed translocations, forensic investigations, and appropriate release of rehabilitated individuals. Incorporating pathogen markers into host assays further enables simultaneous monitoring of disease [60], expanding conservation and management capacity. By uniting these functions into a single, standardised framework, the ideal genomic tool reduces resource demands and enhances the capacity of managers, veterinarians, and researchers to safeguard species persistence in the face of ongoing threats.

The aims of this study were to develop and validate the first integrated, standardised SNP assay for koalas and to demonstrate its applicability across diverse conservation contexts. The assay was designed to unify and expand upon existing genomic resources, including fixed markers from DArTseq and exon-capture genotyping platforms, WGS data, previously characterised fitness-related genes (e.g., immune-associated loci such as Major Histocompatibility Complex (MHC)), and markers targeting prominent koala pathogens (e.g., KoRV). These components were combined into a single, multi-species, comprehensive tool optimised for high information yield and cost efficiency. To ensure accessibility across conservation programs, the assay was designed to be compatible with invasive, non-invasive, and archival sample types. The assay was validated for utility across core applications, including assessment of population diversity and differentiation, parentage assignment, provenance testing, sex determination, and pathogen screening. More broadly, the overarching objective was to establish a scalable genomic framework capable of improving koala conservation outcomes and adaptable for use in other threatened species requiring integrated host (neutral and adaptive)–pathogen genomic monitoring.

## 2. Materials and Methods

### 2.1. Biological Sample Processing

**Figure 1:**
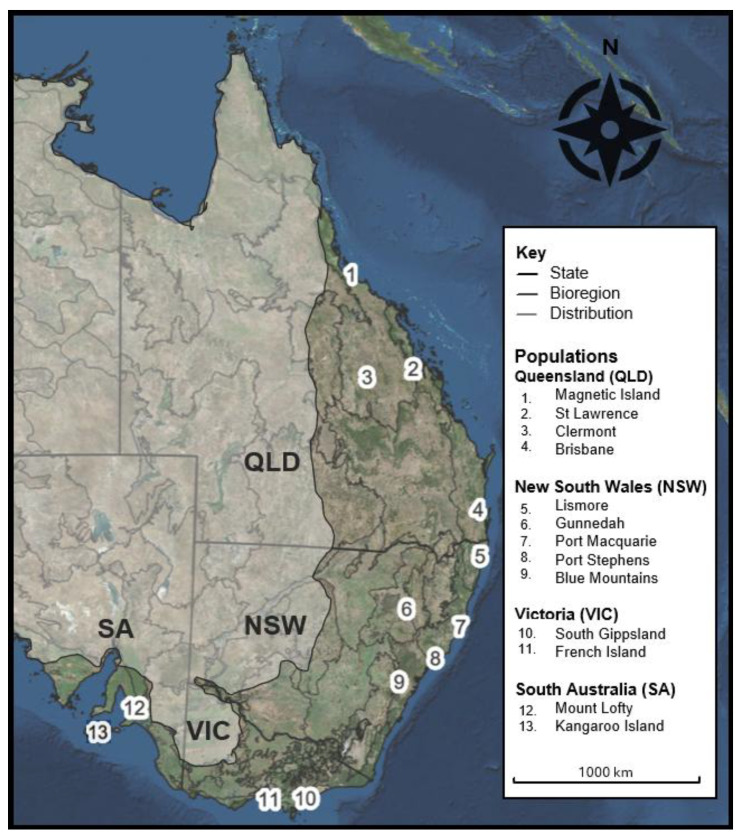
Map of eastern Australia showing state boundaries (black outline) for Queensland (QLD), New South Wales (NSW), Victoria (VIC), and South Australia (SA); Interim Biogeographic Regionalisation for Australia (IBRA v7) bioregions (dark grey outline); and the expected extant koala distribution (non-shaded area). Sampling locations are labelled as follows: 1. Magnetic Island, 2. St Lawrence, 3. Clermont, 4. Brisbane, 5. Lismore, 6. Gunnedah, 7. Port Macquarie, 8. Port Stephens, 9. Blue Mountains, 10. French Island, 11. South Gippsland, 12. Mount Lofty, and 13. Kangaroo Island.

To validate the assay as a standardised genomic tool, 400 koalas from 13 populations, previously characterised in published SNP studies were sourced [13,52]. Populations were selected to represent inhabited major biogeographic regions and genetic lineages spanning the species’ distribution (Figure 1). Biological samples (tissue, blood, scat, hair, and swabs) were either archived or opportunistically collected and obtained from various sample sources (details provided in the Acknowledgements and Supplementary Table S1). Genomic DNA (gDNA) was extracted using a CTAB/chloroform–isoamyl protocol [61], the Bioline ISOLATE II faecal DNA kit [62] or was obtained directly as gDNA extracts [13,52]. All samples were normalised to a working concentration of ~10 ng/µL using a Quantifluor^®^ dsDNA System protocol [63].

### 2.2. Assay Design (SNP Library Preparation)

To develop the standardised, integrated genotyping tool, a custom Allegro^®^ (Tecan Trading, AG, Männedorf, Switzerland) Targeted Genotyping probe-based SNP assay was designed. This NGS technology uses short hybridisation probes to capture and sequence specific user-selected SNPs with high accuracy and throughput, allowing thousands of informative loci to be genotyped simultaneously across many individuals, providing a cost-effective yet scalable platform for conservation genomics [64]. Informative SNPs were identified by mapping loci from published koala genomic datasets (*Koala-fixed*), candidate koala fitness-related gene datasets (*Koala-discovery*) and koala-pathogen gene/genomes (*Pathogen-discovery*), to the latest assembly of the koala reference genome (GCA_002099425.1_phaCin_unsw_v4.1) available at National Centre for Biotechnology Information (NCBI) [65]. These three SNP selection datasets (Supplementary Table S2) were processed as follows:

(1) The *Koala-fixed* dataset consists of SNPs identified from three published koala datasets; (A) DArTseq [13], (B) Exon-capture [52] and (C) WGS-derived [53]. (A) The DArTseq marker set consisted of 4606 SNPs, filtered according to [13], with outliers, sex-linked, parentage and traceability markers identified, and remainder allocated putatively neutral markers [13]. The filtered dataset obtained was mapped to the reference genome using Burrow-Wheeler alignment (BWA) tool [66] and SNP positions were identified within tags with a custom bioawk script. (B) The 2167 exon-capture dataset, filtered according to [52] was obtained and mapped to the reference genome as described above. (C) The WGS-derived marker set was produced by extracting approximately 46,434,864 SNPs from the 387 genomes across the koala distribution, using a modified version of an existing pipeline previously described [67] that employs a consensus approach [68]. These SNPs were then filtered using [BCFtools] [69] with the following thresholds: removing sites with Minor Allele Frequency (MAF) < 0.05 and pruning for Linkage Disequilibrium (LD) by retaining only one SNP per 10 Mb (i.e., one per contig), then retaining only the 1000 SNPs with the highest MAF. In total 5172 SNPs across the three marker sets were kept for downstream analyses.
(2) The *Koala-discovery* dataset was developed by identifying candidate koala fitness-related genes and annotations from the NCBI [65]. Fitness targets included genes linked to immunity and disease (e.g., Chlamydia, viral infections, tumour development (neoplasia)), stress response, metabolism and temperature regulation—reflecting the koala’s broad climatic range. Diet-related genes including the koala-expanded (Cytochrome P450) family (involved in detoxifying diverse, toxic eucalypt compounds), and reproduction-related genes particularly Y -linked (SOX genes from SRY complex) to be utilised for sex-determination. To capture all available sequences for each fitness target a Basic Local Alignment Search Tool (BLAST) v2.11.0 [70] search was performed, and sequences with over 98% similarity and E-value of <0.01 were kept. To identify SNPs for each target, all relevant sequences were aligned using Geneious Prime 20201.1 [71]. A maximum of three SNPs per target gene were retained, prioritising conserved flanking regions (≤75 bp) for probe attachment, selected markers were subsequently mapped to the reference genome as with the *Koala-fixed* dataset.
(3) The *Pathogen-discovery* dataset was developed by identifying major infectious pathogens of the koala with available sequences on NCBI database [65] and that are potentially detectable within a koala biological sample. Gene and genomic sequences for each target pathogen were retrieved using BLAST [70], retaining those with >98% similarity and E-value of <0.01. SNP markers were identified by aligning all sequences to respective reference genome or gene with Geneious Prime 20201.1 [71]. To prevent cross-species contamination, each pathogen reference was mapped against the koala genome to ensure no overlap with host sequences- excluding KoRV which is known to endogenously integrate into the koala genome and was labelled accordingly.

The final *Koala-pathogen* marker set for the custom Allegro assay design was created by integrating the three sub-datasets (*Koala-fixed*, *Koala-discovery*, *Pathogen-discovery*). Duplicate loci, markers with overlapping flanking regions were removed, and anonymous or replicate SNPs were re-labelled to specific gene, if required. To meet the low-density requirements of 4999, the dataset was reduced by randomly removing anonymous SNPs from the WGS-derived dataset.

### 2.3. Assay Development and Genotyping

The custom Allegro^®^ Targeted Genotyping V2 assay was developed with Tecan Genomics and the Australian Genome Research Facility (AGRF) (Melbourne, Australia) using the submitted *koala–pathogen* marker set design. Tecan developed the capture probes for each target SNP [64]. AGRF performed high-throughput sequencing on an Illumina NovaSeq platform (Illumina, Inc., San Diego CA, USA) and then data processing including base calling, demultiplexing, quality control, read alignment to a custom koala–pathogen reference genome, and variant calling using standard pipelines [72]. Final deliverables included raw data and filtered genotyped dataset (now referred to as the ‘*Koala&Pathogen’* dataset), comprising all variants detected within the 150 bp probe region.

### 2.4. Sample Quality Control and Performance

Sample Quality Control (QC) was conducted using the *Koala-fixed* dataset (see below). Two key performance metrics—average read depth and call rate (proportion of non-missing genotypes)—were calculated for each sample with [PLINK v1.9] [73]. Samples with mean read depth < 5 or call rate < 50% were excluded from downstream analyses. Potential batch effects were assessed by comparing performance metrics between replicate samples sequenced on different genotyping plates. Read depth was also used to assess performance across different biological sample types; tissue (ear biopsies and necropsy tissue), blood (whole blood and veterinary by-products), scat (pre-extracted faecal samples), swab (ocular/urogenital swabs collected for *Chlamydia* testing), hair, and historic gDNA (>5 years ago).

### 2.5. SNP Quality Control and Validation

The *Koala&Pathogen* dataset was de-multiplexed by individual samples and the three sub-datasets; *Koala-fixed*, *Koala-discovery* and *Pathogen-discovery*. For each sub-dataset, SNP QC was performed using [PLINK v1.9] [73] and [VCFtools] [74], as described below.

#### 2.5.1. Koala-Fixed Dataset

The *Koala-fixed* dataset, based on previously validated SNPs required minimal filtering. Multiallelic sites were split into biallelic and labelled accordingly, to ensure software compatibility. SNPs were excluded if insertions/deletions (indels), mean read depth ≤ 10, mean call rate ≤ 50%, significant deviation from Hardy-Weinberg Equilibrium (HWE) (*p* < 0.0001), or a total genotyping rate ≤80%.

To validate the *Koala-fixed* dataset with published source datasets, admixture plots with predetermined populations were generated in RStudio [75] using the packages [vcfR] [76], [adegenet] [77], [LEA] [78], [ggplot2] [79], [reshape2] [80]. Analyses were run at equivalent K values (K = 2, 5, 9) to publications [13,52,54] and with comparable populations were included where possible.

#### 2.5.2. Koala-Discovery Dataset

To discover informative fitness-related SNPs, the *Koala-discovery* was filtered more stringently. Retained loci were required to meet the following criteria: biallelic (after multiallelic splitting), non-indel, mean read depth > 10, mean call rate > 50%, independent LD (r^2^ < 0.2) and MAF > 0.01.

The *Koala-discovery* dataset was validated with published MHC class II clustering patterns [81]. Admixture analyses were performed in RStudio [75] using the packages [vcfR] [76], [adegenet] [77], [LEA] [78], ggplot2 [79], [reshape2] [80]. Analyses were conducted at K = 2 for comparability to published data [81] including seven of the same populations (North: Brisbane, Lismore, Gunnedah, Port Mcquarie and Blue Mountains, South: South Gippsland and French Island) (*n* = 153).

Finally, for downstream analysis the *Koala-fixed* and *Koala-discovery* datasets were merged into the *Koala (Fixed & Discovery)* dataset (*n* = 3358 SNPs).

#### 2.5.3. Pathogen-Discovery Dataset

The *Pathogen-discovery* dataset was designed to detect presence of pathogens, therefore underwent minimal QC. Multiallelic sites were split, and only biallelic, non-indel and SNPs with mean read depth ≤ 5 (in one sample) were retained and formulated the *Pathogen-discovery detection* dataset. Using this dataset, individual samples were classified as “potentially positive” for a given pathogen if they had at least one SNP associated with that pathogen. If no SNP found, samples were classified as “assumed negative” for that specific pathogen.

To validate these assay-based classifications, a subset of 47 samples was selected for independent testing via Sanger sequencing [82] with AGRF. This subset included at least six “potentially positive” and at least one “assumed negative” sample per pathogen (where possible). The validation covered seven of the 8 koala pathogens targeted in the assay: KoRV (exogenous (KoRVexo) and endogenous (KoRVend) forms), Chlamydia (*C. pecorum)*, Phascolarctos gammaherpesviruses (PhaHV-1 and -2), Koala papillomavirus (KoAA), and Koala Epstein–Barr virus (KoEBV), as no reads from the assay for *C. pneumoniae* were present in any sample. Target species was confirmed by performing BLAST [70] on returned sanger sequences. To assess diagnostic performance, the results of the assay and sequencing were compared using key performance metrics: sensitivity, specificity, accuracy [83].

### 2.6. Outlier Detection

To identify loci potentially under selection, outlier detection was conducted and validated using three complementary analytical approaches with the *Koala (Fixed & Discovery)* dataset (SNPs = 3358). To minimise bias associated with historical population bottlenecks in southern populations (VIC and SA), only individuals from northern populations (QLD and NSW) were included in the analyses.

(1) Principal Component Adaption (PCAdapt) analysis detects outliers related to local adaption and was performed in RStudio v4.4.0 [75] using the [pcadapt] package [84]. *p*-values were assigned to each locus and adjusted for multiple testing using a False Discovery Rate (FDR) of 0.01. SNPs were considered candidates outliers if *p* < 0.001.
(2) Latent Factor Mixed Model (LFMM) detects associations between SNP variation and environmental predictors while accounting for unobserved population structure through latent factors. Analyses were performed in RStudio v4.4.0 [75] using the packages [adegenet] [77], [LEA] [78], [qvalue] [85], [vegan] [86], [PopGenReport] [86], and [geosphere] [86]. Five environmental variables were tested: mean annual temperature (°C), mean annual rainfall (mm), elevation (m), aridity index, and leaf area index, following [52]. LFMM was run for 5000 iterations with a 10,000 burn-in. *p*-values were adjusted with the Benjamini–Hochberg procedure (FDR 0.01), and loci significantly associated with one or more predictors were retained as candidate outliers.
(3) Redundancy Analysis (RDA) was used to detect multivariate associations between SNP variation and environmental predictors. Analyses were performed in RStudio v4.4.0 [75] using packages [vegan] [87] and [adegenet] [77]. The same five environmental variables tested in LFMM were calculated, and strongly correlated variables were identified using pairwise Spearman’s correlations. Model significance was assessed using permutation tests (*n* = 999). SNP loadings on significant RDA axes were then calculated, and loci with z-scores > 3 were retained as candidate outliers.

To ensure only the most reliable outliers were retained, loci were required to be identified as candidate outliers by all three methods—PCAdapt, LFMM, and RDA. These final outliers were then mapped to the koala reference genome (GCA_002099425.1_phaCin_unsw_v4.1) to identify potential gene associations and infer links to functional traits.

### 2.7. Sex Determination

To assess the assay’s ability to determine sex of samples, an unfiltered candidate *Sex* dataset (*n* = 11,830 ‘DArTseq-Sex’ and ‘Reproduction’ SNPs) was screened for sex-linked markers. Initially sex-association models were created with only sex-known samples (54 Females and 44 Males). SNPs with MAF < 0.05 were removed, and two sex association tests were performed; chi-squared and Fishers exact test with [PLINK v1.9] [73]. Significant SNPs (*p* < 0.05) were retained to formulate a set of candidate sex-linked markers. These were validated by training models with sex-known individuals and applying them to predict the sex of samples with unknown phenotypes.

### 2.8. Parentage Assignment and Provenance

To validate the assay’s ability to assign parentage, the *Koala (Fixed & Discovery)* (*n* = 3358 SNPs, 311 individuals) dataset was examined, including five known mother–offspring pairs (*n* = 10). Prior testing in CERVUS v3.0.7 [88] (Marshall et al., 1998) indicated that ~400 informative SNPs provided reliable parentage assignment. Accordingly, the *Koala (Fixed & Discovery)* dataset was filtered by MAF > 0.35 to generate a *Parentage* dataset (*n* = 399 SNPs). Genotype data were formatted for CERVUS to calculate allele frequencies, heterozygosity, Polymorphic Information Content (PIC), and exclusion probabilities, which informed the likelihood-based parentage simulation. Simulations used 100,000 iterations, a genotyping error rate of 1%, and assumed 90% parental sampling. Offspring were assigned under strict (95%) and relaxed (80%) confidence thresholds, requiring ≥90% loci typed. Known mother–offspring pairs were used to validate assignment accuracy, with logarithm of the odds (LOD) scores and mismatch counts recorded to evaluate marker informativeness and potential genotyping error.

To assess the assay’s ability to assign individuals to their source populations, multiple MAF thresholds were tested to identify the smallest SNP subset capable of reliably distinguishing populations. A filtered subset of the *Koala (Fixed & Discovery)* dataset at MAF > 0.05 was selected as the *Provenance* dataset, as it retained strong discriminatory power while reducing redundancy. Population structure and individual-level assignment patterns were visualised using [NetView] package [89] in RStudio v4.4.0 [75]. Clustering was performed across a range of k-nearest neighbour (kNN) values (5–60), enabling assessment of provenance assignment.

### 2.9. Population Diversity and Differentiation

To validate the standardised assay relative to published koala genomic datasets, population- and individual-level diversity measures were calculated using the *koala (Fixed & Discovery)* dataset (*n* = 3358 SNPs, 311 individuals). Population-level diversity metrics including observed heterozygosity (Ho), expected heterozygosity corrected for sample size (H_Ecorr_), inbreeding coefficient (F_IS_), private alleles (Ap), and rare alleles (Ar; MAF < 0.05)—were estimated in [DiveRsity] [90] using the [*divBasic()]* function the with 10,000 bootstrap iterations. The proportion of polymorphic loci was calculated as the fraction of loci with more than one allele. Individual-level diversity and internal relatedness (IR); standardised multilocus heterozygosity (sMLH) were computed in RStudio v4.4.0 [75] with the [Rhh] package [13,91]. Pairwise relatedness among individuals was estimated in ML-Relate using the maximum likelihood (ML) approach [92], applying a relatedness threshold of > 0.25.

To assess genetic differentiation, pairwise divergence was calculated using the *koala (Fixed & Discovery)* dataset (*n* = 3358 SNPs, 311 individuals). Weir and Cockerham’s (WC) unbiased F_ST_ [93] and Nei’s genetic distance [94] were estimated in R with the packages [adegenet] [77] and [hierfstat] [95]. Loci with missing or monomorphic profiles were excluded. Pairwise F_ST_ values were computed with [*pairwise.WCfst()*], and 95% confidence intervals were obtained via permutation testing with [*boot.ppfst()*] (999 replicates). The resulting F_ST_ matrix was summarised into population-level comparisons using [dplyr] [96], calculating mean F_ST_, confidence intervals, and the number of pairwise comparisons per population pair.

## 3. Results

### 3.1. Assay Design and Development

A total of 4999 target SNPs were submitted for probe design, resulting in an Allegro^®^ Targeted Genotyping V2 assay comprising 5989 probes. Of these 4009 targeted SNPs had a single unique probe and 19.8% were covered by two probes to enhance capture efficiency. Sequencing and variant calling produced 1,011,091 SNPs across the 150 bp probe regions genotype *Koala&Pathogen* dataset. This dataset included 968,664 *Koala-fixed* SNPs—representing > 200-fold inflation compared to the 4695 targeted sites—including 639,610 ‘DArTseq’ (155×), 217,995 ‘exon-capture’ (182×), and 111,059 ‘WGS-derived’ SNPs (297×). It also captured 41,725 *Koala-discovery* SNPs (4262 ‘Diet’, 23,062 ‘Immune’, 2946 ‘Thermoregulation’, 11,455 ‘Reproduction’) and 702 *Pathogen-discovery* SNPs, comprising 398 ‘KoRVexo’, 215 ‘KoRVend’, 42 ‘KoAA’, 3 ‘KoEBV’, 14 ‘PhaHV-1’, 8 ‘PhaHV-2’, 20 ‘*C. pecorum’*, and 2 ‘*C. pneumoniae’* SNPs (Supplementary Table S3).

### 3.2. Sample Quality Control and Performance

**Figure 2:**
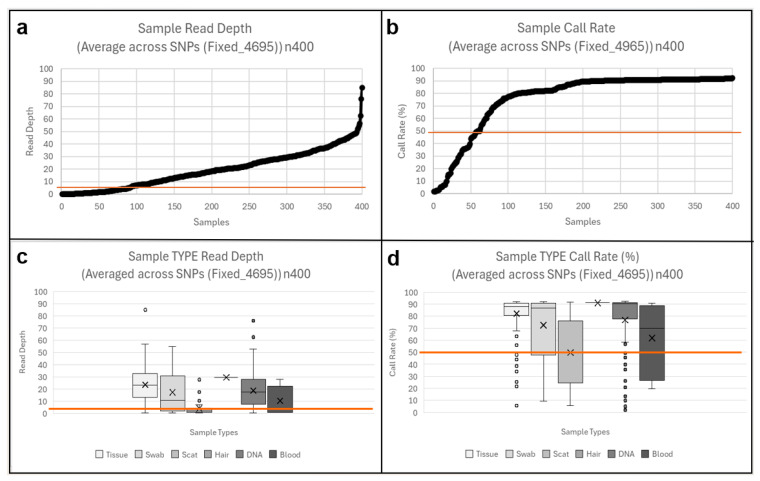
Sample Quality and Performance (n400). TOP: overall sample quality scatterplot (**a**) read depth, (**b**) call rate. BOTTOM: sample type performance boxplots (**c**) read depth, (**d**) call rate. Threshold line (orange) at read depth 5 and call rate 50%.

Of the 400 genotyped samples, 311 (77.8%) passed QC thresholds (mean read depth ≥ 5 and call rate ≥ 50%), visualized with scatterplots (Figure 2). Eighty-seven samples failed due to low coverage or high missingness, and two additional replicates were excluded. No batch effects were detected, with replicate pairs across genotyping plates showing <5% difference in performance metrics.

All biological sample types yielded usable genotyping data, although success rates varied among sample types, as visualised in the boxplots (Figure 2). Tissue samples performed best, with 89% passing QC and showing low variability. The single hair sample passed, as did 80% of historic gDNA. Swab samples showed moderate success (71%) but had the widest variation in depth and call rate. Only 44% of blood samples met thresholds, likely due to suboptimal storage conditions that reduced DNA quality. Scat was the least reliable: only 21% passed QC, typically with very low depth (0–5) but variable call rates (25–75%), reflecting environmental DNA degradation. Overall, while performance differed among sample types, the results confirm that the assay can be applied across both invasive and non-invasive sources.

### 3.3. SNP Quality and Validation

#### 3.3.1. Koala-Fixed Dataset

Of the 4695 exact *Koala-fixed* SNP positions detected in *Koala&Pathogen* dataset (from 4720 submitted), 974 were multiallelic (split into biallelic entries) and 934 indels were removed. Of the remaining loci, 3162 passed the mean read depth (>10) threshold, and 2943 also met the call rate filter (>50%) and were retained. The final dataset included 2018 ‘DArTseq’ SNPs (67.3% of “neutral,” 62.1% of “outlier,” and 56.5% of “sex-linked” loci), 690 ‘exon-capture’ SNPs (65.8%), and 235 ‘WGS-derived’ SNPs (37.2%) (Supplementary Table S3).

**Figure 3:**
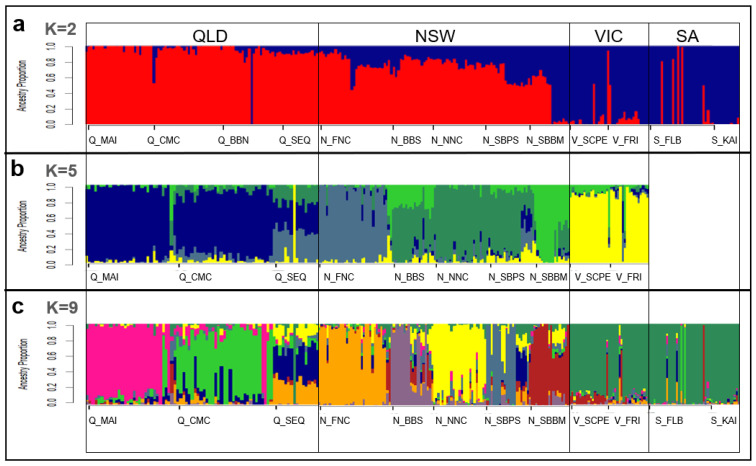
Admixture plots for *Koala-fixed* dataset validation. (**a**) K = 2, (**b**) K = 5, (**c**) K = 9. Colours are aligned with published sources [13,52,54]. Populations (labelled below) (Q_MAI = Magnetic Island, Q_CMC = St Lawrence, Q_BBN = Clermont, Q_SEQ = Brisbane, N_FNC = Lismore, N_BBS = Gunnedah, N_NNC = Port Macquarie, N_SBPS = Port Stephens, N_SBBM = Blue Mountains, V_SCPE = South Gippsland, V_FRI = French Island, S_FLB = Mount Lofty, and S_KAI = Kangaroo Island) and are ordered (left to right) relative to geographical location (north to south). States outlined (QLD, NSW, VIC, SA).

Validation against published koala genomic datasets—including ‘DArTseq’ [13], ‘exon-capture’ [52], and ‘WGS’ [53,54]—showed highly consistent population structure at K = 2, 5, and 9 (Figure 3). At K = 2, a clear north–south divide was observed, reflecting the recognised split between the northern and southern lineages of the species [13,51,52,54]. Major genetic clusters were revealed that correspond to historical biogeographic barriers previously identified across the koala’s range [52,54]. At K = 9, fine-scale structure emerged that aligned with DArTseq [13], including clustering within NSW populations and homogeneity in southern groups. Patterns of admixture in northern QLD were slightly more intermixed than in DArTseq [13] but remained broadly comparable to exon-capture [52] and WGS datasets [54]. Across all comparisons, NSW populations were consistently the most variable, with high admixture in southern NSW, while VIC and SA populations clustered more homogeneously. Overall, these results confirm that the assay robustly reproduces known population structure across the koala’s range and accurately captures both broad- and fine-scale patterns of genetic differentiation.

#### 3.3.2. Koala-Discovery Dataset

Of the 41,725 *Koala-discovery* SNPs, 7048 multiallelic sites were split and 5729 indels removed. After filtering for read depth (>10) and call rate (>80%), 3297 SNPs remained. Additional filtering for LD (r^2^ < 0.2) and MAF (>0.01) yielded 415 high-quality SNPs, comprising 242 immune (44 genes), 41 diet, 31 thermoregulation, and 101 reproduction -related SNPs (Supplementary Table S3).

**Figure 4:**
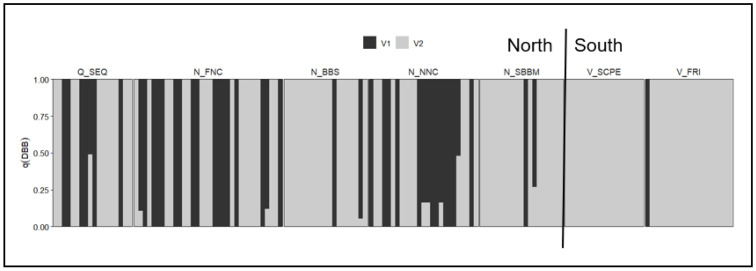
Admixture plot for immune gene MHC II DB-β at K = 2 for seven populations (North: Q_SEQ = Brisbane, N_FNC = Lismore, N_BBS = Gunnedah, N_NNC = Port Macquarie, N_SBPS = Port Stephens, N_SBBM = Blue Mountains. South: V_SCPE = South Gippsland, V_FRI = French Island).

The ‘MHC II DB-β’ subset of the *Koala-discovery* dataset demonstrated the assay’s ability to resolve population-level variation, consistent with the PCR based assessment of diversity of the same gene [81]. Across the distribution (seven populations), analysis at K = 2 revealed a strong north–south divide, with northern populations (Brisbane, Lismore Gunnedah, Port Macquarie and Blue Mountains) exhibiting greater variation than southern populations (South Gippsland and French Island) (Figure 4).

The MHC-associated SNPs exhibited variation among populations, with differences primarily in allele frequencies rather than the presence of unique alleles. This indicates that adaptive diversity is broadly shared but regionally differentiated across the species’ range. These findings highlight the assay’s capacity to detect MHC variation within and between populations, while also validating its inclusion of fitness-related SNPs as informative markers for adaptive genetic monitoring. Further work is needed to explore population patterns at each of the fitness-related genes included in the *Koala-discovery* dataset, as these may reveal locus-specific signals of adaptation not fully captured in this study.

#### 3.3.3. Pathogen-Discovery Dataset

Of 702 Pathogen-discovery SNPs, 93 multiallelic sites were split and 144 indels removed, leaving 651 SNPs (Supplementary Table S3). Of these, 578 (88.8%) had read depth > 5 in at least one individual and were considered “potential pathogen detection SNPs.” These included ‘KoRVexo’ (360 SNPs), ‘KoRVend’ (175), ‘KoAA’ (37), ‘PhaHV-1’ (8), ‘PhaHV-2’ (3), ‘*C. pecorum’* (14), while ‘KoEBV’ and ‘*C. pneumoniae’* had none. Based on these detection loci, 224 of 311 individuals were classified as “potentially positive,” including 78 samples positive for one pathogen, 163 for two, 36 for three, 43 for four, and 3 for five. The remaining 77 had no detectable pathogen SNPs.

Validation of 47 samples with Sanger sequencing showed strong concordance for ‘KoRVexo’, ‘KoRVend’, and ‘KoAA’, indicating reliable detection of these viral agents. In contrast while ‘PhaHV-1/2’ and ‘*C. pecorum’* were predominantly not found in both methods and ‘KoEBV’ was mostly discordant, with positives in Sanger but only one in the assay. BLAST verification confirmed correct species matches for ‘KoRVend’, ‘KoAA’, and ‘KoEBV’. ‘KoRVexo’ sequences mapped to ‘KoRVend’, reflecting the endogenous origin of exogenous infections. Performance metrics indicated the assay had high sensitivity and accuracy (85–100%) for ‘KoRVexo’, ‘KoRVend’, and ‘KoAA’, with ‘KoAA’ achieving perfect specificity (κ = 1) (Supplementary Table S4). ‘KoEBV’ showed poor accuracy (17%) and sensitivity, while ‘PhaHV-1/2’ and ‘*C. pecorum’* results were inconclusive. Overall ‘KoAA’ and ‘KoRV’ performed reliably for screening but remain insufficient for diagnostic use; further validation with larger sample sets is recommended.

### 3.4. Outlier Detection

Outlier detection was performed using the merged *Koala (Fixed & Discovery)* dataset, which comprised 3358 SNPs, including 2943 from the *Koala-fixed* dataset and 415 from the *Koala-discovery* dataset.

(1) PCAdapt analysis only included samples from northern populations (QLD, NSW; *n* = 229), excluding 82 southern individuals, to minimise confounding effects of historical bottlenecks. Pairwise comparisons across the nine northern populations yielded 37 population pairs (Supplementary Figure S1). From this, PCAdapt identified 12,723 candidate outlier SNPs at *p* < 0.01, reduced to 6422 at *p* < 0.001 and finally, 1703 (1618 *Koala-fixed*, 85 *Koala-discovery*) unique candidate outliers after ranking by frequency across pairs and average *p*-value.
(2) LFMM identified 401 candidate SNPs associated with environmental variables (386 *Koala-fixed*, 15 *Koala-discovery*). Histograms of *p*-value distributions showed left-skewed enrichment, indicating non-random associations with environmental factors (Supplementary Figure S1). Manhattan plots revealed several loci with high significance (*−log*_10_*(p)* > 6), suggesting the presence of loci under potential selection.
(3) RDA revealed significant multivariate associations between SNPs and environmental gradients (permutation test: F = 3.84, *p* < 0.001), consistent with literature [52] (Supplementary Figure S1). A total of 536 SNPs (534 *Koala-fixed*, 33 *Koala-discovery*) were significantly correlated with predictors, most strongly with temperature (355 SNPs), followed by LAI (87), elevation (44), aridity (30), and rainfall (22).

Across all three detection methods, 210 high-confidence outlier loci were consistently identified (196 *Koala-fixed* and 14 *Koala-discovery*). The *Koala-fixed* outliers were primarily derived from ‘DArTseq’ (140 SNPs), with smaller contributions from exon-capture (24) and WGS-derived datasets (22), with pre-determined outliers from ‘DArTseq’ representing the largest proportion, as expected [13]. Outliers in the *Koala-discovery* dataset were functionally annotated to genes involved in immune processes (e.g., *CCL20*, *IL1A*, *MHC II UA/DA-α*, *TLR5/7/8/9*), reproduction (*LHB*), and stress response (*MAP2K*), highlighting loci potentially linked to pathogen defence and physiological adaptation. SNPs not identified as outliers in the combined 3358-SNP dataset were classified as putatively neutral and used as a comparative baseline. Overall, these results demonstrate the assay’s capacity to detect loci under selection, reproducing known candidates from previous studies and identifying novel SNPs of potential adaptive significance.

### 3.5. Sex Determination

From the candidate Sex dataset (11,830 SNPs), fifteen were associated with sex (*p* < 0.05), including 6 significant markers (*p* < 0.01) (one ‘DArTseq-Sex’ and 5 ‘Reproduction’; 3 from SOX genes, 1 from GNRH, and 1 LHB). Logistic regression using this subset achieved 85.2% accuracy for females but only 59.1% for males. This likely reflects the unannotated Y-linked regions as the reference genome is female, and the higher representation of X-linked loci (present in both sexes) relative to Y-linked loci. Of 213 individuals with unknown sex, 195 were predicted as female and 18 male. While these results confirm that the assay can identify sex-linked markers and support genetic sex assignment, accuracy—particularly for males—remains limited. Nonetheless, the identified loci represent promising candidates for further investigation into sex-linked traits.

### 3.6. Parentage Assignment and Provenance

Parentage assignment with the assay (*Parentage* dataset (*n* = 399 SNPs)) successfully identified all five known mother–offspring pairs with 95% confidence and no mismatches. Across the full dataset (*n* = 311), exclusion probabilities were moderate—high (non-exclusion probability = 0.88 for first parent), with a mean PIC of 0.37 across loci. Simulated offspring–parent matches yielded positive LOD scores, confirming the reliability of the SNP panel for pedigree inference. While overall resolution was modest—expected given that individuals were sourced from different projects, time points, and locations—these results demonstrate that the assay provides sufficient power for accurate parentage verification and kinship reconstruction in koalas.

**Figure 5:**
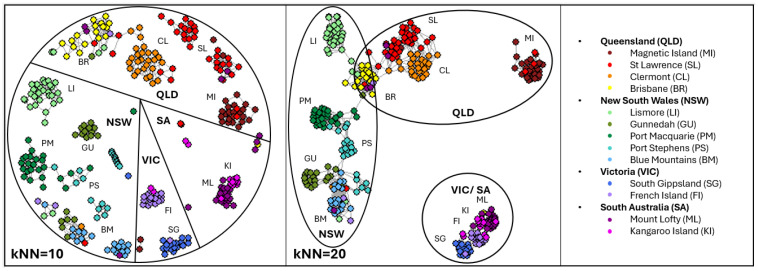
Provenance assignment (*n* = 311) with *Koala (Fixed & Discovery)* MAF > 0.01 (2661 SNPs) visualised with [NetView] at kNN = 10 and 20. Dots are individuals, coloured by pre-defined populations, labelled (see legend) and states outlined.

Provenance assignment was assessed using [NetView] [89] clustering on the *Koala (Fixed & Discovery)* dataset (MAF > 0.05, SNPs = 1808,). Network clustering across multiple k-nearest neighbour (kNN) thresholds (kNN = 10–20) consistently recovered distinct population groupings (Figure 5). The pre-defined population groupings, consistent with those characterised in earlier studies, were also apparent at lower thresholds (kNN = 10). At higher connectivity (kNN = 20), northern populations (St Lawrence, Clermont, Brisbane, Lismore, Gunnedah, Port Macquarie, Port Stephens, Blue Mountains), clusters mirror geographic distribution along the eastern coast, with Magnetic Island forming a distinct, isolated group. The Brisbane population, although located in QLD, is geographically proximate to Lismore and the NSW coast (Port Macquarie), suggesting genetic connectivity between NSW and QLD clusters. Southern populations (South Gippsland, French Island, Mount Lofty, and Kangaroo Island) converged into a single cluster, reflecting the known bottleneck histories. Major regional clusters corresponding to states (QLD, NSW, VIC, and SA) were also evident at higher thresholds. These patterns are consistent with previous DArTseq-based NetView population structuring [13], confirming the assay’s capacity to assign individuals to their population of origin and to resolve both broad- and fine-scale genetic structure across the species’ range.

### 3.7. Population Diversity and Differentiation

Population-level diversity metrics revealed moderate heterozygosity across the 13 populations (H_O_ = 0.09–0.16; H_Ecorr_ = 0.13–0.21), generally consistent with values reported in previous koala genomic studies [13,52,54]. F_IS_ values ranged from –0.07 to 0.20, indicating moderate- low levels of inbreeding or population substructure, with higher F_IS_ observed in southern populations as expected (Table 1). The proportion of polymorphic loci ranged from 29–60%, and rare alleles were present at low frequencies though no private alleles were detected. At the individual level, sMLH and IR varied within and among populations, with southern individuals showing elevated inbreeding signals. While diversity estimates are broadly consistent across datasets, some variation among populations likely reflects uneven sampling, and precision would improve with larger sample sizes. Overall, these diversity patterns align closely with previous findings, with higher genetic diversity in northern populations and reduced diversity in southern and island groups [13,44,51,52,54,97].

Pairwise F_ST_ values showed a moderate to high level of genetic differentiation across populations (Table 2). Overall, estimates from WC unbiased were broadly consistent with those from Nei’s method, though WC values tended to be slightly higher. The lowest divergence was observed between Magnetic Island and St Lawrence (WC F_ST_ = 0.06), expected as St Lawrence has been a consistent source population for Magnetic Island. The highest divergence occurred between Kangaroo Island and Magnetic Island (WC F_ST_ = 0.39), the most geographically distant and both island populations. Despite the absence of private alleles (*Ap* = 0), these patterns indicate that population differentiation is primarily driven by shifts in allele frequencies among shared loci rather than by unique alleles. This suggests historical connectivity followed by restricted contemporary gene flow or drift-driven divergence, consistent with the isolation-by-distance pattern reported in previous publications [13], supporting the assay’s value as a standardised tool for long-term monitoring and conservation management.

## 4. Discussion

This study reports the development and validation of the first standardised, integrated SNP genotyping assay for the koala (*Phascolarctos cinereus*), uniting host and pathogen targets within a single, low-density but highly informative platform. The panel combines putatively neutral and adaptive host loci with pathogen markers, merging SNPs from multiple published datasets—including DArTseq, exon-capture, and WGS data—alongside newly identified variants in fitness-related genes and host-specific pathogen [13,52,54]. By consolidating these resources, the tool reduces reliance on multiple sequencing platforms, providing a cost-effective and scalable approach that simplifies data generation and interpretation for conservation managers, while supporting broader research and veterinary applications [32,33,57].

A key outcome of this work is the establishment of a unified genomic baseline for koalas. Incorporating both neutral and adaptive loci with pathogen targets enables data continuity across projects, laboratories, and timeframes. Such standardisation directly addresses one of the main challenges in wildlife genomics—poor comparability among datasets due to differing platforms or marker sets [57,98,99]. This integrative approach provides a foundation for consistent national-scale monitoring under frameworks such as the *National Koala Recovery Plan* [1], ensuring that future population assessments and management decisions can be supported by directly comparable genomic data.

A defining feature of the assay is its demonstrated performance across a wide range of biological sample types, including invasive (tissue, blood), non-invasive (swab, scat, hair), and archival DNA. Standardising performance across sample types is particularly relevant to koala conservation, where ethical and logistical constraints often limit sampling options [100,101]. Tissue and swab samples yielded the most consistent results, consistent with expectations for high-quality DNA sources. Swabs are widely used for *Chlamydia* testing in koalas [24], highlighting their dual utility for both disease surveillance and host genotyping. Archived blood and historic DNA were moderately successful, most likely reflecting degradation during long-term storage without refrigeration [102]. Scat-derived DNA was the most variable, showing low read depth and inconsistent call rates, consistent with known challenges of contamination and inhibitors in faecal material [100,103,104]. All sample types produced usable genotypes despite limitations, underscoring the robustness of the panel and its value for population-scale monitoring, including hard-to-sample wild individuals [105,106].

The assay recovered over 90% of *Koala-fixed* SNPs from previous genomic platforms, ensuring backward compatibility with legacy datasets while confirming its robustness for standardised analyses. Population structure results recaptured known patterns of north–south divergence (QLD and NSW versus VIC and SA), and regional substructure, consistent with prior genomic studies [13,52,53,54]. The inclusion of fitness-related genes provided further biological insight, with adaptive loci detected in immune genes (e.g., *MHC I UA; II DA-α*, *CCL20*, *IL1A*, *TLR-5;-7;-8;-9*) involved in pathogen recognition and defence against *Chlamydia* and KoRV [23,107]. Additionally, reproductive genes such as *LHB*, associated with induced ovulation and fertility in marsupials [108]; and metabolic or stress-response genes (*MAP2K* and related pathways) potentially linked to environmental resilience and aridity tolerance [109].

Outlier analyses using PCAdapt, LFMM, and RDA identified 210 high-confidence SNPs, including both previously reported outliers [13] and novel loci associated with immune and environmental functions. These results closely align with earlier adaptive signals detected in the exon-capture dataset [52], reinforcing validation of the assay. Together, these findings demonstrate the assay’s value as a standardised tool for tracking both neutral and adaptive variation in koalas, supporting long-term conservation and management [33,57].

A novel dimension of this assay is the integration of host-specific pathogen SNPs within the same genotyping framework, a first for a non-model wildlife species. This allows for simultaneous assessment of host genomic diversity and disease surveillance—an emerging priority in conservation biology [110,111,112]. Validation via Sanger sequencing [113] confirmed high concordance for KoRV and KoAA loci, supporting their use in routine screening and complementing existing diagnostic approaches [23,114,115,116]. Although limited positive controls prevented comprehensive validation of additional targets (e.g., KoEBV, *PhaHV*, *C. pecorum*), these preliminary results demonstrate the feasibility of joint host–pathogen monitoring, offering a scalable model for wildlife health surveillance. Integrating host and pathogen markers in a single workflow represents a significant step towards holistic, ecosystem-level genetic monitoring in threatened species [117,118].

The assay also proved effective for several core conservation applications, including sex determination, parentage assignment and provenance testing [32]. Sex-linked SNPs achieved moderate predictive accuracy (~70%), particularly valuable for unsexed juveniles, archived material, and non-invasive samples [119]. Limited reference data for the Y chromosome constrained the identification of strictly male-linked loci, but these preliminary markers provide a useful foundation for studies of sex-biased dispersal and disease susceptibility [24,120]. Parentage analyses using high-MAF subsets correctly identified all known mother–offspring pairs with ≥90% confidence, confirming suitability for pedigree validation in captive and semi-managed populations [121]. Genetic parentage assignment remains essential for avoiding inbreeding and maintaining accurate records in breeding programs, especially where pouch joey misidentification can occur during handling [46,122]. Provenance analysis based on population structure and network clustering (NetView) reliably resolved geographic differentiation among broader regions (QLD, NSW and VIC and SA), with finer subdivision to populations at higher resolution—mirroring previous studies [13,51]. Provenance testing has direct applications in wildlife rescue, translocation, reintroduction, and forensic investigations, where understanding population origin supports informed management decisions [98,123]. Together these findings highlight the assay’s versatility, providing a standardised and scalable genomic tool applicable across research, management, and clinical contexts.

Patterns of genetic diversity and differentiation were broadly consistent with previous koala genomic studies [13,16,52,54]. Moderate heterozygosity (H_O_ = 0.09–0.16; H_Ecorr_ = 0.13–0.21) and low to moderate inbreeding coefficients indicate limited inbreeding for most populations and moderate within-population variation, in agreement with estimates from DArTseq and exon-capture datasets [13,52]. The proportion of polymorphic loci was high (>50%) across all northern populations (QLD and NSW) but markedly lower in southern populations (of VIC and SA), reflecting historical bottlenecks and reduced diversity in the latter regions. Further, the absence of private alleles and lower heterozygosity in southern and island populations reflect long-term isolation, founder effects, and reduced effective population sizes associated with past translocations and demographic contractions [10,14]. Elevated IR and reduced sMLH in these southern groups further support restricted gene flow and increased local relatedness.

Pairwise F_ST_ values (0.06–0.39) indicated moderate to strong differentiation among regions, consistent with an isolation-by-distance pattern and historical fragmentation documented in previous genomic analyses [13,52]. The lowest divergence between Magnetic Island and St Lawrence (F_ST_ = 0.06) corresponds with records identifying St Lawrence as the source population for Magnetic Island, whereas the highest divergence between Kangaroo Island and Magnetic Island (F_ST_ = 0.39) reflects extreme geographic isolation and independent demographic histories. Together, these results demonstrate that regional structure and founder effects continue to shape koala genetic diversity, highlighting the importance of maintaining connectivity and implementing ongoing genomic monitoring to preserve adaptive potential across the species’ distribution.

### 4.1. Limitations

Several limitations remain. The highly repetitive nature of the koala genome reduced on-target read depth in some probes, introducing uncertainty into the dataset, which could be addressed through probe redesign as more complete genome assemblies become available. Further, incomplete annotation of the koala reference genome—particularly the Y chromosome—restricts interpretation of some adaptive and sex-linked loci. DNA from scat and other degraded sources continues to constrain retrospective analyses, though improvements in collection and storage protocols can help mitigate this issue. Finally, pathogen validation was limited by the availability of positive and negative controls, highlighting the need for larger and more balanced datasets to refine estimates of diagnostic performance.

### 4.2. Future Directions

Future applications of this assay could include a fine scale, detailed assessment of highly diverse and complex population structure (e.g., NSW) to help unravel local connectivity and adaptive variation. A broad scale examination, incorporating underrepresented populations such as inland NSW and northern QLD, enabling more comprehensive understanding across the species’ distribution. Establishing a national koala–pathogen genomic database and integrating host–pathogen–environment data will facilitate GWAS to identify drivers of disease susceptibility and local adaptation. Expanding the framework to include gene expression and epigenetic markers would further enhance resolution of health, fitness, and adaptive responses. Finally, the flexible design of this standardised SNP assay makes it adaptable to other threatened species facing similar conservation challenges, extending its relevance beyond koalas.

## 5. Conclusions

In conclusion this study delivers a novel, standardised SNP assay for the koala, integrating host and pathogen genomic targets within a single, low-density yet highly informative platform. Validated across multiple sample types and conservation applications, it reliably reproduces known patterns of diversity and structure, identifies adaptive loci, and supports pathogen surveillance. By combining host and pathogen targets into one unified genotyping framework, the assay bridges research, management, and clinical applications, facilitating long-term, standardised monitoring across the species’ range. Its versatility extends to sexing, parentage, and provenance testing, providing a cost-effective and scalable genomic tool for evidence-based conservation of one of Australia’s most iconic species. Moreover, this integrative framework demonstrates how standardised genomic tools can be adapted for other threatened taxa, supporting adaptive management, health surveillance, and resilience assessment under changing environmental conditions [33,57,58].

## Figures and Tables

**Table 1:** Diversity Indices table per population. Indices include average observed heterozygosity (H_O_), average expected heterozygosity corrected for sample size (H_Ecorr_), inbreeding coefficient (F_IS_), number of private alleles (*Ap*), and number of rare alleles (Ar; MAF), standardized multilocus heterozygosity (sMLH) and internal relatedness (IR). Based on 10,000 bootstraps with the *Koala (Fixed & Discovery)* dataset.

State	No.	Population Name	Bioregion	n	H_O_	He_corr_	% PL	F_IS_	AvF_ST_	Ap	rare A	Ar	sMLH	IR
QLD	1	Magnetic Island	Q_MAI	31	0.14	0.20	55.05	0.06	0.22	0	0.04	1.37	1.02	0.60
	2	St Lawrence	Q_CMC	33	0.15	0.19	56.42	0.03	0.18	0	0.02	1.44	1.01	0.60
	3	Clermont	Q_BBN	28	0.16	0.21	57.55	0.02	0.18	0	0.03	1.39	1.01	0.61
	4	Southeast QLD	Q_SEQ	19	0.16	0.19	60.32	0.05	0.15	0	0.00	1.50	1.01	0.60
NSW	5	Lismore	N_FNC	34	0.14	0.19	59.01	0.09	0.20	0	0.02	1.40	1.00	0.61
	6	Gunnedah	N_BBS	19	0.15	0.21	53.26	0.07	0.18	0	0.04	1.39	1.00	0.62
	7	Port Macquarie	N_NNC	25	0.15	0.20	55.94	0.02	0.18	0	0.02	1.43	0.99	0.61
	8	Port Stephens	N_SBPS	21	0.15	0.20	56.56	0.03	0.17	0	0.03	1.43	1.00	0.62
	9	Blue Mountains	N_SBBM	19	0.15	0.20	52.46	0.03	0.18	0	0.02	1.42	0.99	0.61
VIC	10	South Gippsland	V_SCPE	17	0.11	0.16	35.67	−0.03	0.24	0	0.04	1.24	0.98	0.63
	11	French Island	V_FRI	20	0.10	0.16	48.73	0.22	0.20	0	0.12	1.24	0.99	0.63
SA	12	Mount Lofty	S_FLB	30	0.10	0.16	55.22	0.19	0.22	0	0.02	1.36	0.99	0.63
	13	Kangaroo Island	S_KAI	15	0.09	0.13	29.09	−0.07	0.26	0	0.02	1.21	0.99	0.65
			**TOTAL**	**311**										

**Table 2:** F_ST_ values per population with *n* > 10. Calculated using: (top-right) Weir and Cockerman’s unbiased approach based on 999 permutations [93]; (bottom-left) Nei’s unbiased genetic distance [94] *koala (Fixed & Discovery)* dataset.

	Q_MAI	Q_CMC	Q_BBN	Q_SEQ	N_FNC	N_BBS	N_NNC	N_SBPS	N_SBBM	V_SCPE	V_FRI	S_FLB	S_KAI
**Q_MAI**		0.05	0.08	0.12	0.18	0.20	0.18	0.18	0.23	0.37	0.32	0.33	0.39
**Q_CMC**	0.06		0.04	0.07	0.13	0.16	0.14	0.14	0.19	0.32	0.28	0.29	0.35
**Q_BBN**	0.09	0.05		0.07	0.13	0.15	0.15	0.13	0.18	0.32	0.27	0.29	0.35
**Q_SEQ**	0.13	0.07	0.07		0.08	0.11	0.10	0.08	0.14	0.28	0.23	0.24	0.31
**N_FNC**	0.20	0.15	0.15	0.08		0.16	0.15	0.14	0.18	0.33	0.29	0.30	0.36
**N_BBS**	0.22	0.17	0.17	0.13	0.17		0.09	0.09	0.10	0.29	0.24	0.26	0.32
**N_NNC**	0.20	0.16	0.16	0.11	0.16	0.10		0.07	0.13	0.30	0.26	0.28	0.34
**N_SBPS**	0.20	0.15	0.15	0.09	0.16	0.10	0.08		0.11	0.27	0.23	0.24	0.30
**N_SBBM**	0.25	0.21	0.20	0.15	0.20	0.12	0.14	0.13		0.24	0.19	0.22	0.28
**V_SCPE**	0.40	0.37	0.36	0.31	0.37	0.32	0.34	0.30	0.26		0.05	0.07	0.10
**V_FRI**	0.36	0.32	0.31	0.25	0.32	0.27	0.29	0.25	0.21	0.05		0.03	0.05
**S_FLB**	0.36	0.32	0.32	0.26	0.32	0.28	0.30	0.26	0.23	0.08	0.03		0.04
**S_KAI**	0.45	0.41	0.40	0.34	0.41	0.36	0.38	0.34	0.31	0.11	0.05	0.04	

## Data Availability

Restrictions apply to the datasets—The datasets presented in this article are not readily available because the data are part of an ongoing study. Requests to access the datasets should be directed to Lily Donnelly.

## Supplementary Materials

The following supporting information can be downloaded at: https://www.mdpi.com/article/10.3390/ani15233375/s1, Figure: Supplementary Figure S1. Outlier analysis results. (a) PCAdapt pairs (northern populations only) with number of unique outliers per population pair in top right triangle. (b) LFMM *p* value (Histogram and Manhattan) plots for each environmental predictor (top to bottom *p* = 1–7). (c) RDA Environmental Predictor (mean annual Temperature (°C), mean annual. Rainfall (mm), Elevation (m), Aridity Index and Leaf Area Index) matrix. Bottom left triangle = scatterplot of variable, top right triangle = Spearman correlation coefficient between variables, diagonal = histograms/density plots of each variable’s distribution; Table: Supplementary Table S1. Metadata for 400 koalas from 13 populations, including population, sex, and biological sample type. DNA extraction and storage methods varied by sample type: previously extracted gDNA; tissue, blood, swab, and hair extracted with a modified CTAB/chloroform–isoamyl method in this study; and scat DNA extracted with the Bioline^®^ Faecal DNA Kit [62]. Blood was obtained stored in ethanol, all other ‘types’ were obtained frozen, stored in TE/H_2_O; Supplementary Table S2. SNP selection table including; *Koala-fixed* (SNP datasets) *Koala-discovery* (candidate fitness genes), and *Pathogen-discovery* (pathogen genes & genomes), with GenBank (NCBI) Accession number and sequence/dataset sources; Supplementary Table S3. Number of SNPs for each category; submitted in design, genotyped, filtered; Supplementary Table S4. Validation of pathogen detection results for Assay and Sanger Sequencing of 47 samples, for each pathogen (Koala retrovirus KoRV (exo and endo), *Chlamydia pecorum (C. pecorum)*, Koala gammaherpesviruses (PhaHV-1 & -2), Koala papillomavirus (KoAA), and Koala Epstein Barr Virus (KoEBV)), by population. Supplementary Table S5. Total filtered, Outlier and Neutral SNPs Koala (Fixed & Discovery) *n* = 3358 SNPs, 218 individuals (north) by SNP category and subcategory, found with PCAadapt, *p* > 0.001, FDR 0.01, with ‘northern’ populations only (‘southern’ expected to have population bottleneck skew data).

## Abbreviations

The following abbreviations are used in this manuscript:

AGRF	Australian Genome Research Facility
Ap	Private alleles
Ar	Rare Alleles
BLAST	Basic Local Alignment Search Tool
BWA	Burrow–Wheeler alignment
C. pecoum	Chlamydia pecorum
DArTseq	Diversity Arrays Technology sequencing
EPBC	Environmental Protection and Biodiversity Conservation
FDR	False Discovery Rate
F_IS_	Inbreeding coefficient
gDNA	Genomic DNA
GWAS	Genome-wide association studies
H_Ecorr_	Expected heterozygosity (corrected for sample size)
H_O_	Observed heterozygosity
HWE	Hardy–Weinberg Equilibrium
IR	Internal Relatedness
IUCN	International Union of Conservation of Nature
kNN	k-nearest neighbour
KoAA	Koala papillomavirus
KoEBV	Koala Epstein–Barr Virus
KoRV	Koala Retrovirus
KoRVend	Endogenous koala retrovirus
KoRVexo	Exogenous koala retrovirus
LD	Linkage Disequilibrium
LFMM	Latent Factor Mixed Model
LOD	logarithm of the odds
MAF	Minor allele Frequency
MHC	Major Histocompatibility Complex
ML	Maximum likelihood
mtDNA	mitochondrial DNA
NCBI	National Centre for Biotechnology Information
NGS	Next-Generation Sequencing
NSW	New South Wales
PCAdapt	Principal Component Adaption
PhaHV	Phascolarctos gammaherpesviruses
PIC	Polymorphic Information Content
QC	Quality Control
QLD	Queensland
RAPD	Randomly Amplified Polymorphic DNA
RDA	Redundancy Analysis
SA	South Australia
sMLH	standardised multilocus heterozygosity
SNP	Single-Nucleotide Polymorphism
VIC	Victoria
WC	Weir and Cockerham’s
WGS	Whole-genome sequencing
