# Supplementary material for: Development and Validation of a Standardised Genomic Tool for Conservation Management of the Koala (Phascolarctos cinereus)
Source: Animals (Basel). 2025 Nov 21;15(23):3375. doi: 10.3390/ani15233375 (PMC12691185; doi:10.3390/ani15233375)
Supplement: Supplementary file 1 [file animals-15-03375-s001.zip › animals-3938233-supplementary.pdf]

6. Supplementary Tables and Figures (with captions)

Table S1. Metadata for 400 koalas from 13 populations, including population, sex, and biological sample type. DNA extraction and storage methods varied by sample type: previously extracted gDNA; tissue, blood, swab, and hair extracted with a modified CTAB/chloroform–isoamyl method in this study; and scat DNA extracted with the Bioline® Faecal DNA Kit {Bioline, #539}. Blood was obtained stored in ethanol, all other 'types' were obtained frozen , stored in TE/H_2O).

Population					Sex			Sample Type					
No.	State	Name	Code	n	F	M	U	DNA	Tissue	Blood	Swab	Scat	Hair
1	QLD	Magnetic Island	Q_MAI	36	5	6	25	19	17	0	0	0	0
2		St Lawrence	Q_CMC	36	8	14	14	13	23	0	0	0	0
3		Clermont	Q_BBN	34	19	13	2	0	34	0	0	0	0
4		Brisbane	Q_SEQ	36	2	2	32	5	12	0	0	19	0
5	NSW	Lismore	N_FNC	36	7	5	24	13	21	0	0	2	0
6		Gunnedah	N_BBS	20	0	0	20	0	20	0	0	0	0
7		Port Macquarie	N_NNC	36	0	0	36	27	0	0	0	8	1
8		Port Stephens	N_SBPS	25	14	3	8	1	10	0	14	0	0
9		Blue Mountains	N_SBBM	35	5	6	24	19	16	0	0	0	0
10	VIC	South Gippsland	V_SCPE	20	0	0	20	16	4	0	0	0	0
11		French Island	V_FRI	30	0	0	30	30	0	0	0	0	0
12	SA	Mount Lofty	S_FLB	35	0	0	35	28	7	0	0	0	0
13		Kangaroo Island	S_KAI	21	0	0	21	11	1	9	0	0	0
				**Sub Total**	60	49	291	182	165	9	14	29	1
			**Total**	**400**		**Sex**	**400**					**TYPE**	**400**

Table S2. SNP selection table including; *Koala-fixed* (SNP datasets) *Koala-discovery* (candidate fitness genes), and *Pathogen-discovery* (pathogen genes & genomes), with GenBank (NCBI) Accession number and sequence/ dataset sources.

Type	Category	Sub Category		Reference	GenBank Accession
	DArTseq	DArTseq		Kjeldsen et al., 2019	
	Exon-capture	Exon		Lott et al., 2022	
Koala - Fixed	WGS-derived	WGS		Hogg et al., 2023	
		CLEC3	C-type Lectin, Family 3, B	Johnson et al, 2018	XM_020987344.1, XM_020987343.1, XM_020987343.1
		CLEC5	C-type Lectin, Family 5, B	Johnson et al, 2018	XM_020998113.1, XM_020998119.1, XM_020998118.1, XM_020998117.1, XM_020998116.1, XM_020998115.1, XM_020998114.1, XM_020998120.1, XM_020998121.1,
		CYP450	Cytochrome P450	Johnson et al, 2018	XM_020980812.1, EU_581952, EU_581951.1
		OLF	Olfactory receptor	Johnson et al, 2018	AF_262715.1, XM_020969278.1, AF_262699.1
		TAS2R	taste receptor	Johnson et al, 2018	XM_020972141, XM_0270972140
		VIR1	Vomeronasal receptor type-1	Johnson et al, 2018	XM_020978858, XM_020978856.1, XM_020978859.1, XM_020978857.1, XM_020978860.1
	Diet	VIR3	Vomeronasal receptor type-3	Johnson et al, 2018	XM_020978841.1, XM_020978839.1
		BMP2	bone morphogenetic protein 2	Johnson et al, 2018	XM_020985637.1, XM_027857527.1
		BMPK	Inducible kinase	Johnson et al, 2018	XM_020988363.1, XM_027854613.1
		BTLA	B and T Lymphocyte	Johnson et al, 2018	XM_021007174.1, XR_002327868.1, XM_021007173.1
		CASP	Caspase 2	Johnson et al, 2018	XM_020997945.1, XM_020997944.1, XM_020997942.1, XM_020997943.1,
		CCL20	C-C motif chemokine ligand 20	Johnson et al, 2018	XM_020970978.1, XM_020970977.1, XM_020985548.1,
		CD8	CD8	Maher et al., 2014, Johnson et al., 2018	XM_020969485.1, KF_731655.1, KF_731656.1
		COX2	Cytochrome oxidase type 2	Ngo, et al., 2003, Johnson et al., 2018	XM_021002463.1, XM_021002461.1, XM_021002465.1, XM_021002464.1, AF_463454.1, AF_463453.1
		DExH	DExH box helicase 58	Abts, et al., 2015, Johnson et al., 2018	XM_020972773.1, XM_020972772.1, XM_002321734.1, KP_792548.1
		IFNy	Interferon gamma	Mathew, et al., 2013, Maher et al., 2014, Johnson et al., 2018	XM_020990282.1, XM_020990283.1, KC_894757.1, KF_731657.1
		IL10	Interleukin 10	Mathew, et al., 2013, Maher et al., 2014, Johnson et al., 2018	XM_021002936.1, JX_845581.1, KF_731659.1
		IL17	Interleukin 17a	Mathew, et al., 2014, Johnson et al, 2018	KJ_174517, XM_020993976.1
		IL1a	Interleukin 1a	Johnson et al, 2018	XM_020963580.1
		IL2a	Interleukin 2a	Johnson et al, 2018	XM_020973669.1, XM_020973668.1, XM_020973667.1, XM_020973665.1, XM_020973664.1, XM_020973666.1,
		IL2b	Interleukin 2b	Johnson et al, 2018	XM_020982521.1, XM_020982520.1, XM_020982522.1, XM_020982519.1, XM_020982518.1,
		IL4	Interleukin 4	Maher et al., 2014, Johnson et al., 2018	XM_020991403.1, KF_731658.1
		IL6	Interleukin 6	Maher et al., 2014, Johnson et al., 2018	XM_020993366.1, KF_731661.1
		MHCIIDAa	Major Histocompatabilty Complex II - DA-a	Johnson et al, 2018, Lau et al., 2013, Abts et al., 2015, Jobbins et al., 2012	JX109917, JX109918, KP792566, XR_002323139, XM_020980723, JX109927, JX109928, JX109929, JX109930, JX109931, JX109932, JX109933, JX109934, HM748767, HM748768, HM748776, HM748777, HM748778, HM748779, HM748780, HM748781, HM748782, HM748783, HM748784, HM748785, HM748786, HM748787, HM748788, HM748789
		MHCIIDAb	Major Histocompatabilty Complex II - DA-b	Johnson et al, 2018, Lau et al., 2013, 2014, Abts et al., 2015, Robbins et al., 2020, Quigley et al., 2019	JX514157, JX514158, JX514153, JX514154, JX514155, JX514156 , KP792559, MG8577484, MG8577485, MT321034, MT321039, MT321040, MT321041, MT321042, MT321043 MG8577486, MG8577487, MG8577488, MG8577489, XM_020974195
		MHCIIDBa	Major Histocompatabilty Complex II - DB-a	Johnson et al, 2018, Lau et al., 2013, Abts et al., 2015	JX109919, JX109920, JX109921, KP792561, XM020974137, XM020974138, XM020974227
		MHCIIDBb	Major Histocompatabilty Complex II - DB-b	Johnson et al, 2018, Lau et al., 2013, 2014, Abts et al., 2015, Robbins et al., 2020	JX109922, JX109923, JX109924, JX109925, JX109926, JX514151, JX514152, KP792560, MT321035, MT321044, MT321045, MT321046, MT321047, MT321048, MT321049, MT321050, MT321051, MT321052, MT321053, MT321054, MT321055, MT321056, MT321057, MT321058, MT321059, MT321060, XM_020974333, XM_020974334
		MHCIIDMb	Major Histocompatabilty Complex II - DM-b	Johnson et al, 2018, Abts et al., 2015, Robbins et al., 2020	KP792557, MT321061, MT321062, MT321063, MT321064, XM_020994004, XM_020994005
		MHCIUA	Major Histocompatabilty Complex I - U-a	Johnson et al, 2018, Robbins et al., 2020, Houlden et al., 1996c, Cheng et al., 2018	MT321017, MT321018, MT321019, MT321020, MT321021, MT321022, MT321023, MT321024, MT321025, U33812, MT321026, MT321027, MT321028, MT321029, MT321030, KY612373, KY612374, KY612375, KY612376, KY612377, KY612378, KY612379
		MHCIUB	Major Histocompatabilty Complex I - U-b	Johnson et al, 2018, Robbins et al., 2020, Abts et al., 2015, Cheng et al., 2018	KP792563, KY612380, KY612381, KY612382, KY612383, KY612384, MT321031, MT321032, MT321033,
		MHCIUC	Major Histocompatabilty Complex I - U-c	Johnson et al, 2018, Cheng et al., 2018	XM_020974196, KY612385, KY612386, KY612387, KY612388, KY612386, KY612386, KY612387, KY612388, KY612389, KY612390, KY612391, KY612392, KY612393
		NKC	Natural Killer Cell triggering receptor	Johnson et al., 2018	XM_020999177.1, XM_020999174.1, XM_020999176.1, XM_020999175.1, XM_020999178.1,
		PTGER4	Prostaglandin E receptor -4	Johnson et al., 2018	XM_020996229.1, XM_020996221.1
		S100	S100 calcium binding protein B	Johnson et al., 2018	XM_020973442.1
		TCRa	T cell receptor - a	Johnson et al., 2018	XR_002320566.1
		TLR13	Toll Like Receptor - 13	Cui et al., 2015	KP334160, KP334161
		TLR16	Toll Like Receptor - 1.6	Johnson et al., 2018, Abts, et al., 2015, Cui et al., 2015	KM816880, KM816881, KP792540, XM021007478, XM021007479, XM021007480, XM021007481, XM021007483, XM021007484
		TLR2	Toll Like Receptor - 2	Johnson et al., 2018, Abts, et al., 2015	KP792545, XM021007691, XM021007693, XM021007694, XM021007695, XM021007696, XM021007697, XM021007698, XM021007699, XM021007700
		TLR3	Toll Like Receptor - 3	Johnson et al., 2018, Abts, et al., 2015, Cui et al., 2015	KP792547, KM816856, KM816857, KM816858, KM816859, XM_020990604
		TLR4	Toll Like Receptor - 4	Johnson et al., 2018, Abts, et al., 2015, Cui et al., 2015	KM816860, KM816861, KM816862, KM816863, KM816864, KM816865, KM816866, KM816867, KM816868, KM816869, KM816870, KM816871, KP792551, XM020982198, XM020982199, XM020982200, XM020982201, XM020982203, XM020982204, XM020982205
		TLR5	Toll Like Receptor - 5	Johnson et al., 2018, Abts, et al., 2015, Cui et al., 2015	KM816872, KM816873, KM816874, KM816875, KM816876, KM816877, KM816878, KM816879, KP792550, XM020991810, XM020991816, XM020991823
		TLR7	Toll Like Receptor - 7	Johnson et al., 2018, Abts, et al., 2015, Cui et al., 2015	KM816882, KM816883, KM816884, KM816885, KP792558, XM021007491
		TLR8	Toll Like Receptor - 8	Johnson et al., 2018, Abts, et al., 2015, Cui et al., 2015	KM816886, KM816887, KM816888, KM816889, KM816890, KP792567, XM020971571
		TLR9	Toll Like Receptor - 9	Johnson et al., 2018, Abts, et al., 2015, Cui et al., 2015	KM816891, KM816892, KM816893, KM816894, KM816895, KM816896, KM816897, KM816898, KM816899, KM816900, KP792555, XM020964590
		TMP5	Transmembrane protein 5	Johnson et al., 2018	XM_020980795.1, XM_020980327.1, XM_0209980795.1, XM_0209980379.1
	Immune	ZNF420	zince finger protein 420-like	Johnson et al., 2018	XM_020971821.1, XM_020971850.1, XM_020971851.1
		GAPDH	glyceraldehyde-3-phosphate dehydrogenase	Johnson et al., 2018, Maher et al., 2015	XM_020994140.1, KF_31660.1
		GSTO1	glutathione S-transferase omega 1	Johnson et al., 2018	XM_020987967.1, XM_020987968.1, XM_020987967.1
		INS	Insulin	Johnson et al., 2018	XM_020970881.1
		NADH2	dehydrogenase subunit 3	Osborne et la., 2002	AF_425985.1
		NADH4	dehydrogenase subunit 4	Johnson et al., 2018	XM_020993643.1
	Metabolism	NADH5	dehydrogenase subunit 5	Wedrowicz, et al., 2017	KY979230.1, KY979222.1, KY979221.1
		ERR1	estrogen receptor 1	Johnson et al., 2018	XM_020986619.1, XM_020992451.1, XM_020992450.1, XM_020992449.1 , XM_020992448.1, XM_020992447.1
		ERRA	estrogen receptor - alpha	Johnson et al., 2018	XM_020967961.1, XM_020967960.1
		ERRG	estrogen receptor - gamma	Johnson et al., 2018	XM_020982268.1, XM_020982267.1, XM_020982266.1, XM_020982263.1, XM_020982265.1, XM_020982261.1
		GNRH1	gonadotropin releasing hormone - 1	Johnson et al., 2018, Busby, et al., 2015	XM_021001216.1, KJ_191755.1
		GNRH2	gonadotropin releasing hormone - 2	Johnson et al., 2018, Busby, et al., 2015	XM_021005521.1, KJ_191756.1
		LHB	Lutenizing hormone B	Johnson et al., 2018, Busby, et al., 2015	XM_021003428.1, KJ_191760.1
		LLP	Late lactation protein	Johnson et al., 2018	XM_020981388.1, XM_020981390.1, XM_020981372.1
		SOX01	SRY-box - 1	Johnson et al., 2018	XM_020965121.1
		SOX02	SRY-box - 2	Johnson et al., 2018	XM_020980825.1
		SOX03	SRY-box - 3	Johnson et al., 2018	XM_020971786.1
		SOX04	SRY-box - 4	Johnson et al., 2018	XM_020986834.1
		SOX05	SRY-box - 5	Johnson et al., 2018	XM_020994276.1, XM_020994275.1
		SOX06	SRY-box - 6	Johnson et al., 2018	XM_021005404.1
		SOX07	SRY-box - 7	Johnson et al., 2018	XM_020988907.1
		SOX08	SRY-box - 8	Johnson et al., 2018	XM_021006895
		SOX09	SRY-box - 9	Johnson et al., 2018	XM_021002623.1
		SOX10	SRY-box - 10	Johnson et al., 2018	XM_020982580.1
		SOX11	SRY-box - 11	Johnson et al., 2018	XM_020992722.1
		SOX12	SRY-box - 12	Johnson et al., 2018	XM_020990539.1
		SOX13	SRY-box - 13	Johnson et al., 2018	XM_021003127.1
		SOX14	SRY-box - 14	Johnson et al., 2018	XM_020986152.1
	Reproduction	SOX15	SRY-box - 15	Johnson et al., 2018	XM_020966486.1
		HIF3	hypoxia inducible factor 3	Johnson et al., 2018	XM_020978436.1
		HSP90	heat shock protein 90	Johnson et al., 2018	XM_020993853.1, XM_02993852.1
		HSTF3	heat shock transcription protein 3	Johnson et al., 2018	XM_02997113.1
		MAPK2	mitogen-activated protein kinase 2	Johnson et al., 2018	XM_020968167.1
	Thermoregulation	MAPK7	mitogen-activated protein kinase 7	Johnson et al., 2018	XM_020978135.1
		P53	tumour protein p53	Johnson et al., 2018	XM_021009839.1, XM_021009841.1,
		RASSF1	RAS association family 1	Johnson et al., 2018	XM_020999297.1, XM_02099296, XM_020999295.1
Koala - Discovery	Tumour	TNFa	tumour necrosis factor alpha	Johnson et al., 2018	XM_020974214.1, JX_845580.1
		C. pecorum	Chlamydia pecorum	Jelonick, et al., 2020	KT_223767.1, KT_223766.1, KT_223768.1, KT_223781.1, KT_22376=77.1, KT_223780.1, KT_223774.1, KT_223771.1,
	Chlamydia	C. pneumonia	Chlamydia pneumoniae	Glassick et al., 1996	U_56925.1
	Papilloma	KoAA	Koala papillomavirus isolate KoAA1	Antonsson et al., 2016	DQ_464069.1
	Epstein Barr virus	KoEBV	Phascolarctos cinereus Epstein-Barr virus induced 3 (EBI3), mRNA	Johnson et la., 2018	XM_020971434.1
		KoRV end	Koala retrovirus - endogenous DNA	Hanger et al,. 2000, Tarlintont, et al., 2014	KF_786286, KF_786280, KF_786284, KF_786282, KF_786283, KF_786281, KF_786285, AB_721500, NC_039228
	Retrovirus	KoRV exo	Koala retrovirus - exogenous RNA	Xu e tal., 2013, Shojima, et al., 2013, Shimode et al, 2014	KC_779547.1
		PhaHV-1	phascolartid hherpesvirus 1	Vaz et al., 2011	JN_585829.1
Pathogen - Discovery	Herpesvirus	PhaHV-2	phascolartid hherpesvirus 2	Vaz et al., 2012	JQ_996387.1

Table S3. Number of SNPs for each category; submitted in design, genotyped, filtered.

Type	Category	Sub Category	Design	Genotype	Filtered
		Neutral	2952	2886	1942
		Outliers	64	66	41
	DArTseq	Sex	62	62	35
	Exon-capture	Exon	1036	1049	690
	WGS-derived	WGS	606	632	235
Koala - Fixed	**TOTAL**		**4720**	**4695**	**2943**
		CLEC3	3	541	1
		CLEC5	3	567	6
		CYP450	3	590	9
		OLF	3	655	12
		TAS2R	3	672	4
		VIR1	3	572	3
	Diet	VIR3	3	665	6
		BMP2	3	550	2
		BMPK	3	361	1
		BTLA	3	514	8
		CASP	2	208	4
		CCL20	3	492	8
		CD8	2	272	3
		COX2	3	0	0
		DExH	3	609	9
		IFNy	3	305	1
		IL10	3	501	4
		IL17	3	581	2
		IL1a	3	662	3
		IL2a	3	580	2
		IL2b	3	529	4
		IL4	3	500	6
		IL6	3	475	4
		MHCIIDAa	3	495	10
		MHCIIDAb	2	0	0
		MHCIIDBa	3	667	12
		MHCIIDBb	2	338	14
		MHCIIDMb	3	679	4
		MHCIUA	3	306	23
		MHCIUB	3	379	9
		MHCIUC	3	371	12
		NKC	3	642	1
		PTGER4	3	662	8
		S100	3	403	3
		TCRa	3	660	7
		TLR13	3	649	2
		TLR16	3	515	2
		TLR2	3	649	5
		TLR3	3	674	5
		TLR4	3	695	5
		TLR5	3	661	7
		TLR7	3	685	6
		TLR8	3	636	3
		TLR9	3	621	6
		TMP5	3	386	7
	Immune	ZNF420	3	631	5
		GAPDH	3	576	7
		GSTO1	3	563	3
		INS	3	473	4
		NADH2	3	0	0
		NADH4	4	178	2
	Metabolism	NADH5	3	0	0
		ERR1	3	580	5
		ERRA	3	460	3
		ERRG	3	538	3
		GNRH1	3	348	7
		GNRH2	3	388	5
		LHB	3	363	3
		LLP	3	0	0
		SOX01	3	514	5
		SOX02	3	437	5
		SOX03	3	538	3
		SOX04	3	589	7
		SOX05	3	610	4
		SOX06	3	675	10
		SOX07	3	534	5
		SOX08	3	664	3
		SOX09	3	640	4
		SOX10	3	601	3
		SOX11	3	643	5
		SOX12	3	564	8
		SOX13	3	641	1
		SOX14	3	571	8
	Reproduction	SOX15	3	557	4
		HIF3	3	415	4
		HSP90	3	608	4
		HSTF3	3	658	9
		MAPK2	3	648	10
	Thermoregulation	MAPK7	3	617	4
		P53	3	573	6
		RASSF1	3	601	1
Koala -	Tumour	TNFa	3	555	2
Discovery	**TOTAL**		**243**	**41725**	**415**
		C. pecorum	3	20	212
	Chlamydia	C. pneumonia	3	2	360
	Papilloma	KoAA	2	42	38
	Epstein Barr virus	KoEBV	3	3	2
		KoRV end	10	215	14
	Retrovirus	KoRV exo	10	398	6
		PhaHV-1	3	14	17
Pathogen -	Herpesvirus	PhaHV-2	2	8	2
Discovery	**TOTAL**		**36**	**702**	**651**
**TOTAL**			**4999**	**47122**	**4009**

Table S4. Validation of pathogen detection results for Assay and Sanger Sequencing of 47 samples, for each pathogen (Koala retrovirus KoRV (exo and endo), *Chlamydia pecorum (C.pecorum)*, Koala gammaherpesviruses (PhaHV -1 & -2), Koala papillomavirus (KoAA), and Koala Epstein Barr Virus (KoEBV)), by population.

		Allegro Assay		Sanger Sequencing		Performance			
**Pathogen**	**n**	**Pos**	**Neg**	**Pos**	**Neg**	**Accuracy**	**Sensitivit**	**Specificit**	**Precision**
Koala retrovirus - exogenous (KoRVexo)	7	7	0	7	0	1	1	NA	1
Koala retrovirus - endogenous (KoRVend	7	6	1	7	0	0.857	0.857	NA	1
Phascolarctid herpesvirus - 1 (PhaHV1)	6	0	6	0	6	1	NA	1	NA
Phascolarctid herpesvirus - 2 (PhaHV2)	7	0	7	0	7	1	NA	1	NA
Koala Papillomavirus (KoAA)	7	5	2	5	2	1	1	1	1
Koala Epstein Barr Virus (KoEBV)	6	1	5	6	0	0.167	0.167	NA	1
Chlamydia pecorum (C. pecorum)	7	4	3	0	7	0.429	NA	0.429	0

S5. Total filtered, Outlier and Neutral SNPs (Koala (Fixed & Discovery) n = 3,358 SNPs, 218 individuals (north) by SNP category and subcategory, found with PCAadapt, $p > 0.001$, FDR 0.01, with 'northern' populations only ('southern' expected to have population bottleneck skew data).

	RDA		PCAadapt		LFMM		Shared	
**Type**	**Categroy**	**No. of outliers**	**Categroy**	**No. of outliers**	**Categroy**	**No. of outliers**	**Categroy**	**No. of outliers**
	DSEQ_NEU	357	DSEQ_NEU	654	DSEQ_NEU	274	DSEQ_NEU	132
	DSEQ_OUT	18	DSEQ_OUT	27	DSEQ_OUT	20	DSEQ_OUT	13
	DSEQ_SEX	11	DSEQ_SEX	17	DSEQ_SEX	9	DSEQ_SEX	5
	EXON_CAP	107	EXON_CAP	213	EXON_CAP	77	EXON_CAP	24
	WGS_AWS	41	WGS_AWS	73	WGS_AWS	43	WGS_AWS	22
**Fixed**	**Total**	**534**	**Total**	**984**	**Total**	**423**	**Total**	**196**
	DIET_CLEC5	2	DIET_CLEC5	3	DIET_CLEC5	1		0
		0	DIET_OLF	2		0		0
		0	DIET_TAS2R	1		0		0
	DIET_VIR3	2	DIET_VIR3	1		0		0
		0	IMMU_BMPK	1		0		0
	IMMU_CCL20	1	IMMU_CCL20	1	IMMU_CCL20	1	IMMU_CCL20	1
	IMMU_CD8	1	IMMU_CD8	1		0		0
	IMMU_IL17	1	IMMU_IL17	1		0		0
	IMMU_IL1a	2	IMMU_IL1a	2	IMMU_IL1a	2	IMMU_IL1a	1
	IMMU_IL2b	1	IMMU_IL2b	2		0		0
		0	IMMU_IL4	2	IMMU_IL4	1		0
	IMMU_MHCIIDAa	3	IMMU_MHCIIDAa	4	IMMU_MHCIIDAa	3	IMMU_MHCIIDAa	3
		0	IMMU_MHCIIDBa	4		0		0
		0	IMMU_MHCIIDBb	5	IMMU_MHCIIDBb	2		0
	IMMU_MHCIIDMb	1	IMMU_MHCIIDMb	3	IMMU_MHCIIDMb	2		0
	IMMU_MHCIUA	1	IMMU_MHCIUA	9	IMMU_MHCIUA	2	IMMU_MHCIUA	1
		0	IMMU_MHCIUB	2		0		0
		0	IMMU_MHCIUC	3		0		0
	IMMU_S100	1		0		0		0
		0	IMMU_TLR13	1		0		0
	IMMU_TLR2	2		0	IMMU_TLR2	2		0
		0	IMMU_TLR3	1		0		0
	IMMU_TLR4	1	IMMU_TLR4	1	IMMU_TLR4	1		0
	IMMU_TLR5	3	IMMU_TLR5	2	IMMU_TLR5	2	IMMU_TLR5	2
	IMMU_TLR7	1	IMMU_TLR7	2	IMMU_TLR7	1	IMMU_TLR7	1
	IMMU_TLR8	2	IMMU_TLR8	1	IMMU_TLR8	1	IMMU_TLR8	1
	IMMU_TLR9	2	IMMU_TLR9	3	IMMU_TLR9	2	IMMU_TLR9	2
		0	IMMU_TMP5	1		0		0
	IMMU_ZNF420	1	IMMU_ZNF420	1		0		0
	METAB_INS	1	METAB_INS	1		0		0
		0	METAB_NADH4	1		0		0
		0	REPRO_ERRG	3		0		0
		0	REPRO_GNRH1	2		0		0
		0	REPRO_GNRH2	1	REPRO_GNRH2	1		0
	REPRO_LHB	1	REPRO_LHB	1	REPRO_LHB	1	REPRO_LHB	1
		0	REPRO_SOX02	2		0		0
		0	REPRO_SOX04	3	REPRO_SOX04	1		0
		0	REPRO_SOX05	1		0		0
		0	REPRO_SOX08	1		0		0
		0	REPRO_SOX09	1		0		0
		0	REPRO_SOX10	1		0		0
	REPRO_SOX15	1		0		0		0
		0	THERM_HIF3	2		0		0
		0	THERM_HSTF3	2	THERM_HSTF3	1		0
	THERM_MAPK2	1	THERM_MAPK2	3	THERM_MAPK2	3	THERM_MAPK2	1
	TUMR_P53	1	TUMR_P53	3		0		0
		0	TUMR_TNFa	1		0		0
**Discovery**	Total	33	Total	89	Total	30	Total	14
**Grand Total**		**567**	**Grand Total**	**1073**	**Grand Total**	**453**	**Grand Total**	**210**

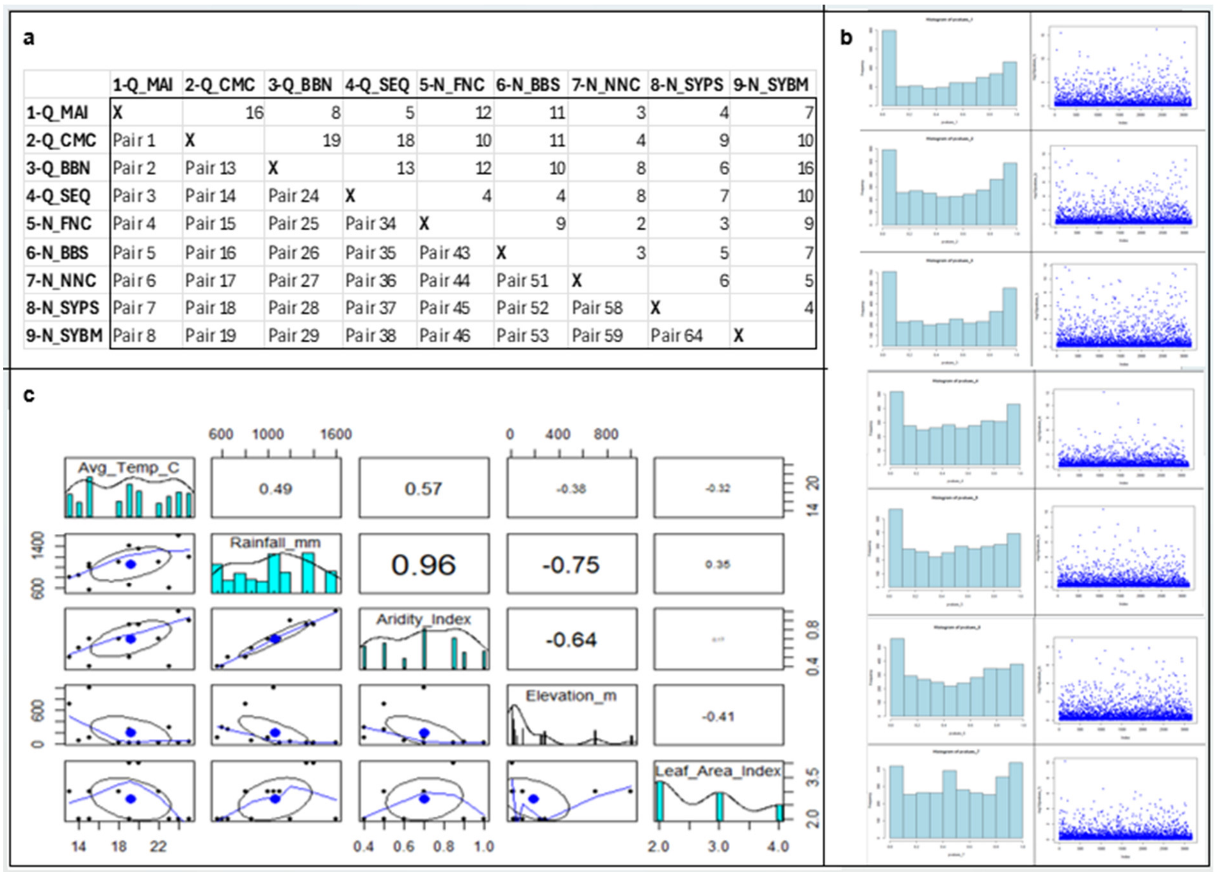

a

	1-Q_MAI	2-Q_CMC	3-Q_BBN	4-Q_SEQ	5-N_FNC	6-N_BBS	7-N_NNC	8-N_SYPS	9-N_SYBM
1-Q_MAI	X	16	8	5	12	11	3	4	7
2-Q_CMC	Pair 1	X	19	18	10	11	4	9	10
3-Q_BBN	Pair 2	Pair 13	X	13	12	10	8	6	16
4-Q_SEQ	Pair 3	Pair 14	Pair 24	X	4	4	8	7	10
5-N_FNC	Pair 4	Pair 15	Pair 25	Pair 34	X	9	2	3	9
6-N_BBS	Pair 5	Pair 16	Pair 26	Pair 35	Pair 43	X	3	5	7
7-N_NNC	Pair 6	Pair 17	Pair 27	Pair 36	Pair 44	Pair 51	X	6	5
8-N_SYPS	Pair 7	Pair 18	Pair 28	Pair 37	Pair 45	Pair 52	Pair 58	X	4
9-N_SYBM	Pair 8	Pair 19	Pair 29	Pair 38	Pair 46	Pair 53	Pair 59	Pair 64	X

Supplementary Figure 1. Outlier analysis results. a) PCAdapt pairs (northern populations only) with number of unique outliers per population pair in top right triangle. b) LFMM *p* value (Histogram and Manhattan) plots for each environmental predictor (top to bottom *p* = 1 – 7). c) RDA Environmental Predictor (mean annual Temperature (°C), mean annual. Rainfall (mm), Elevation (m), Aridity Index and Leaf Area Index) matrix. Bottom left triangle = scatterplot of variable, top right triangle = Spearman correlation coefficient between variables, diagonal = histograms/ density plots of each variable's distribution
